# Considerations for Choice of Cranioplasty Material for Pediatric Patients

**DOI:** 10.1159/000528543

**Published:** 2022-12-07

**Authors:** Megan E.H. Still, Sonja Samant, Abraham Alvarado, Dan Neal, Lance S. Governale, Jessica A. Ching

**Affiliations:** ^a^Division of Pediatric Neurosurgery, Department of Neurosurgery, University of Florida, Gainesville, Florida, USA; ^b^Division of Plastic and Reconstructive Surgery, University of North Carolina, Chapel Hill, North Carolina, USA; ^c^Division of Plastic and Reconstructive Surgery, Department of Surgery, University of Florida, Gainesville, Florida, USA; ^d^Craniofacial Center, UF Health Shands Children's Hospital, Gainesville, Florida, USA

**Keywords:** Pediatric cranioplasty, Craniectomy, Autologous bone, Alloplastic cranioplasty

## Abstract

**Introduction:**

Optimal material and timing of cranioplasty in the pediatric population continue to be debated. Autologous and alloplastic materials have various indications for use and risk factors for complications.

**Methods:**

A single-center retrospective cohort study was undertaken of all pediatric patients who underwent cranioplasty with any material from 1991–2021.

**Results:**

149 cranioplasty implants were included. Younger age (6 years old or under), a diagnosis of craniosynostosis as reason for implant, use of autologous bone, and shorter times to cranioplasty were predictive of need for revision surgery. No factors studied had a statistically significant impact on rate of removal of implant at time of revision surgery.

**Conclusion:**

Autologous and alloplastic cranioplasty materials both have good outcomes with low rates of revision surgery in the pediatric population. Alloplastic implants may be considered in the setting of infection as reason for craniectomy given the lower rate of revision surgery and need for removal. Patients with craniosynostosis as reason for cranioplasty have a higher risk of requiring revision or additional surgeries, regardless of implant used.

## Introduction

Cranioplasty procedures have been conducted for centuries to correct defects from prior craniectomy procedures or for trauma, craniosynostosis, congenital syndromes, tumors, and infection [1]. For adult patients undergoing surgical repair of a cranial defect, autologous bone is considered the gold standard when available. However, determining the ideal cranioplasty implant for the pediatric population presents a challenge due to different patient factors including continued skull growth, bone healing, and the potential for implant migration, tissue reaction, breakage, congenital conditions, and different infection risks [2].

Autologous bone continues to be thought the best option for cranioplasty due to the benefits of the potential to grow with the patient's growing cranium. It seems a safe option for the patient as it has perfect histocompatibility and can fuse quickly with the adjacent bone [3]. However, there still exists a risk of infection and a higher incidence of bone resorption in the pediatric population, with the highest incidence being seen in patients younger than 7 years old [2]. In the case of severe bone loss or insufficient donor site bone availability, autologous bone grafting is not an option [4].

When autologous bone is not available or viable, alloplastic materials such as metallic, acrylics, and ceramics may be used. Among metallic materials, the most commonly used is titanium mesh, which is known for having excellent strength and good resistance to infection but can be difficult to work with and mold to the skull [5]. Other options such as hydroxyapatite, polymethyl methacrylate (PMMA), polyetheretherketone (PEEK), and other acrylics have been used with various pros, cons, and long-term outcomes demonstrated in published literature [4, 5, 6, 7].

Although there are numerous materials available for cranioplasties, there remains a lack of data on complication risk or long-term outcomes for their use in the pediatric population. This study evaluates a pediatric cohort who underwent calvarial defect reconstruction with autologous bone, PEEK implants, hydroxyapatite, acrylic, or titanium implants and assesses the risk factors for reoperation or implant removal based on patient factors and implant type.

## Methods

Institutional data were retrospectively reviewed for all pediatric patients who underwent cranioplasty from 1991 through July 2021. Patients were excluded if they underwent cranial vault remodeling only, suturectomy for craniosynostosis or minimally invasive synostosis repair, or elevation of depressed skull fracture with autologous replacement of fractured fragments only. Primary outcomes evaluated were need for revision surgery and removal of bone flap. Variables of interest included age of patient when undergoing cranioplasty, gender, underlying diagnosis requiring cranioplasty, and timing of cranioplasty in regard to initial craniectomy or skull defect. The category of craniosynostosis as a reason for cranioplasty included both primary craniosynostosis repair if it involved advancement of a local bony flap and/or implants and those who underwent prior synostectomy or synostosis repair who presented with a secondary skull defect. This category was separated from patients who underwent cranioplasty for skull defects from other prior surgeries due to the potential differences in underlying bony morphology or physiology that prompted craniosynostosis in the first place.

Fisher's exact test and Wilcoxon test were used to determine which factors had a statistically significant impact on rates of complications and reoperation. *p* < 0.05 was considered statistically significant.

## Results

240 patients who underwent cranioplasty at our institution were identified, and 149 patients met inclusion criteria. The number of patients who underwent autologous, bone cement, PEEK, titanium, PMMA, hydroxyapatite, acrylic, or combination cranioplasty can be seen in Table 1. The majority of patients in our cohort (64.4%) underwent autologous cranioplasty.

**Fig. 1:**
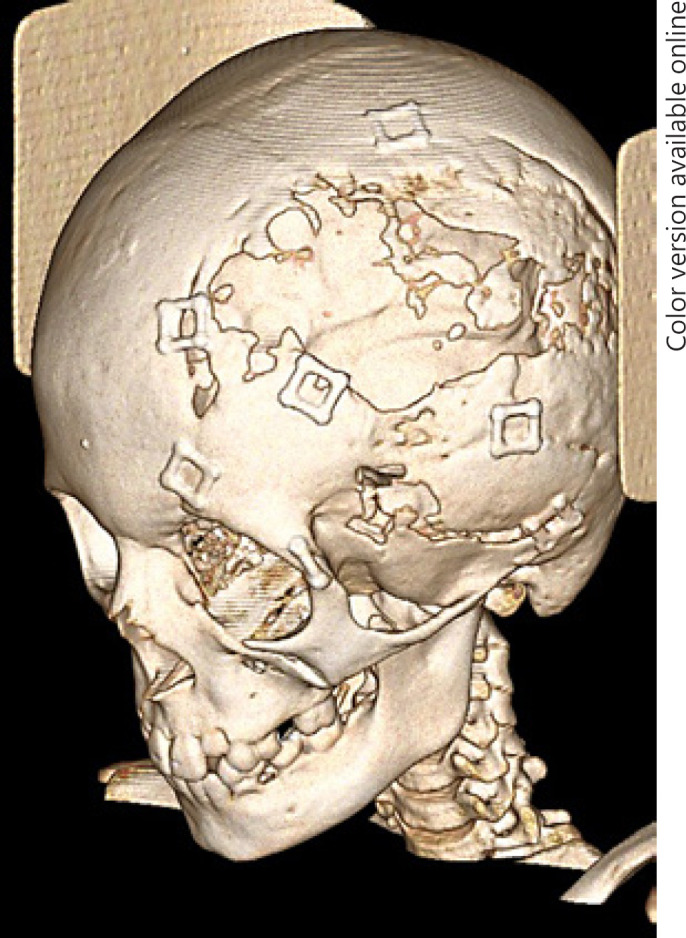
Example of bony reabsorption necessitating revision of cranioplasty.

Forty-one patients (27.5%) underwent revision surgery due to cranioplasty fracture after trauma, bony reabsorption, infection, and craniosynostosis with subsequent bony defects or continued craniofacial reconstruction. A representative example of bony reabsorption which required revision surgery is demonstrated in Figure 1. Age 6 or under, craniosynostosis as an underlying reason for cranioplasty, and use of autologous bone for cranioplasty were statistically significant predictors of need for revision surgery (Table 1). When compared with all other implant types combined, autologous cranioplasty had a higher rate of need for revision surgery (*p* = 0.004).

Seventeen patients (11.4%) underwent removal of implant during revision surgery (Table 2). No factors studied were statistically significant predictors of need for implant removal during revision surgery, though craniosynostosis as an underlying diagnosis trended towards significance.

Eighty-eight patients (56.4%) underwent immediate cranioplasty within the same operation as the craniectomy, typically for tumor resection, cosmetic revision, secondary skull defect, or repair of growing skull fracture. Of these, 34 patients (38.6%) required removal of their implant at a later time. Shorter time to implant placement was a statistically significant predictor of both need for revision surgery and for removal of implant at the time of surgery. Of all patients that required revision of their bone flap, the average time was 0.43 months (SD 25.0; median 0 [IQR 0, 4] [range 0, 185]), as compared with the 8.5-month (SD 1.1; median 0 [IQR 0, 0)] [range 0, 5]) average time for patients not requiring revision (*p* = 0.0001). For those who required removal of bone implant at time of revision, the average time to cranioplasty was 0.15 months (SD 0.42; median 0 [IQR 0, 0] (range 0, 1.5]) as compared to 7.1 months (SD 22.8; median 0 [IQR 0, 3] [range 0, 185]) in those who did not require removal of their implant (*p* = 0.006). This trend remained true when patients who underwent cranioplasty on day zero of surgery were removed from analysis.

## Discussion

There is a paucity of data regarding the long-term outcomes of different cranioplasty material in the pediatric population. This retrospective study evaluated reoperation and removal of bone flap rates in a pediatric cohort who underwent calvarial reconstruction for a variety of underlying conditions. In this study, craniosynostosis as an underlying diagnosis or reason for cranioplasty was a statistically significant predictor of need for revision of implant and trended towards significance as a reason for removal of implant at time of revision. Younger age (6 years old or less) was an additional predictor of need for revision surgery. Autologous bone had the highest rate of reoperation (82.9% of all revisions). Synthetic materials had minimal reoperation, with the PEEK implant having none. Shorter time to cranioplasty was also a predictor of need for revision surgery and removal of implant.

Autologous bone has historically been considered an ideal implant option for the pediatric population due to its capacity to grow with the growing skull once it is revascularized and the overall low reported infection rate [8, 9]. The most common donor site when the autologous flap is damaged or unavailable for reimplantation is split calvarial graft from another cranial site or split rib graft. However, autologous implants have a high risk of resorption, particularly in children younger than 7 years old or if rib graft is used. Additionally, there is pain and risk of morbidities at the donor site, limited bone available to harvest in small or young patients, and can prove to be difficult to contour [2, 6, 7, 10, 11]. A recent systematic review demonstrated that published reabsorption rates range from 30 to 80% and a 20% graft failure rate, particularly when banked bone was used [6, 12, 13, 14, 15]. In that report, banked autologous implant had the highest rate of complications and graft failure rate, while fresh autologous bone had one of the lowest rates of infection, surgical site complications, and graft failure rates [6]. This demonstrates well the complexities of successful cranioplasty in the pediatric population, showing that not only the material but also the circumstances and timing of implantation have a significant impact on outcomes.

In our study, the majority of patients underwent autologous cranioplasty, and the majority of patients who required revision surgery and/or removal of their implant also had autologous cranioplasties. All patients but one who underwent revision surgery due to infection had an autologous implant, and no patients with alloplastic cranioplasty experienced reabsorption or fracture of implant. Additionally, 13 of 27 (48.1%) patients who underwent cranioplasty for infection received autologous implant, but 100% of the patients who required revision surgery after cranioplasty for infection had autologous implants. None of the patients who underwent alloplastic cranioplasty for infection required revision surgery or removal of implant. This indicates that autologous cranioplasty may result in increased complications and need for revision surgery in patients who require cranioplasty for intracranial infection.

As the use of synthetic materials is gaining traction, current literature is expanding on the different types and their benefits. Among metallic materials, the most common is titanium, which is known for having excellent strength but can be difficult to manipulate intraoperatively [5]. Benefits of titanium mesh include limitless supply, lower operative time without need for obtaining graft, immediate protection without need for bony growth and integration, no donor site morbidity, and low rates of infection and graft failure [2, 6]. Titanium implants were very well tolerated in our patient population, with only 1 of 13 patients requiring revision surgery with removal of the implant, in that instance due to fracture of the implant and scalp necrosis from a halo vest.

Hydroxyapatite is a porous material designed to allow osteoblast migration through the implant for improved integration with the host bone and thus offers excellent cosmetic results [12]. Some reports demonstrate its use results in the lowest rate of reoperation. However, hydroxyapatite implants can be brittle and be more likely to break with minor trauma, especially prior to bone integration, and are not recommended for use in children under 7 years old due to skull growth and increased risk of inflammatory response to the polymer [5, 10, 12]. Hydroxyapatite was not frequently used but appeared to be successful in our patient cohort. 19 patients (12.8%) underwent hydroxyapatite implantation, with three requiring revision surgery and none requiring removal of their flap at the time of revision. All 3 patients had revisions due to bony defects and ongoing cosmetic repair for craniofacial defects from syndromic craniosynostosis, and none of our patients experienced fracture of the implant.

PMMA and PEEK are mixed alternatives based on acrylic compounds. PMMA implants tend to be relatively more malleable, but long-term studies show that they can become brittle, have high infection rates, and have exothermic properties that can be damaging to the overlying tissue with time [5]. PEEK more closely reflects the strength and elasticity of bone, requiring drilling to mold to defects, and can be re-sterilized for future procedures. Both also have commercially available options for preoperative CT-guided mold-based patient-specific implant creation, allowing for lock-in-key fit reducing operative time, blood loss, and potentially anesthetic effects [1, 5]. This makes them well suited for a myriad of complex defects if time is not of the essence. These overall favorable findings were found to be consistent in our patient population, though evaluation is limited by the low rate of use: only 1 patient who underwent cranioplasty with PMMA underwent revision surgery due to superficial skin infection, and none required removal of implant. No patients who underwent PEEK implantation required revision surgery.

While continued growth of the skull has always been a consideration in pediatric cranioplasty, one study demonstrated no defects due to skull growth with any alloplastic implant at 82 months of follow-up, indicating this may be a less prevalent problem than once believed, particularly in children older than six [12]. There are also varying conflicting reports regarding the frequency of infection with each type of implant, importance of timing from craniectomy to cranioplasty, and underlying condition (tumor vs. trauma vs. infection) on rates of complications, and need for additional surgeries, further confounding the decision-making process.

Overall, our findings indicate that alloplastic cranioplasty material appears to be an appropriate and viable alternative in the pediatric population, particularly if autograft has previously failed or is unavailable. Of those who required revision surgery with alloplastic implants, the reason for revision was overwhelmingly due to ongoing craniofacial reconstruction for patients with craniofacial syndromes rather than complications directly related to the implant itself or material used. Conversely, in our cohort, revision surgery for patients with autologous cranioplasty were more frequently due to surgical site complications, such as infection (most often requiring wound washout and debridement rather than flap removal) and bony shifts/pullout with hardware prominence. Thus, in our patient cohort, alloplastic cranioplasty may be a better first-line option with fewer implant-related complications.

Appropriate timing of cranioplasty after craniectomy is also a highly debated subject. While many reports indicate there is no significant difference in cognitive outcomes between cranioplasties performed within 3 months of craniectomy and after, other complications such as hydrocephalus and wound breakdown contribute to outcomes data and may be more significantly impacted by timing [16, 17, 18]. Though our data indicate that shorter time to cranioplasty is a predictive factor for the complications studied, this result is confounded by the inclusion of implants placed on day zero of surgery, such as cranioplasty placed after removal of a skull tumor, many of which consisted of repair of secondary skull defects after previous cranioplasty or craniofacial surgery. In these cases, it would not be possible to delay surgery as the surgery is not to repair an iatrogenic skull defect but one that developed later after a previous surgery. A sub-group analysis after removal of patients who underwent secondary cranioplasty (implant on surgery day zero in this study) also revealed a shorter time to implant as a risk factor for need for revision surgery and removal of implant. This indicates that time to cranioplasty after craniectomy may impact rates of revision surgery, and delay of cranioplasty at least a few weeks after index surgery, if feasible in the clinical context, should be considered when appropriate.

The data reported here indicate that alloplastic materials are a safe alternative to autologous cranioplasty and may have fewer implant-related complications requiring revision surgery or implant removal in the pediatric population. While the majority of patients underwent autologous cranioplasty with good results, those who received alloplastic implants had a low rate of revision surgery and removal, and those who did require revision surgery frequently did so due to syndromic progression rather than implant complications. Of note, our data indicate that patients with craniosynostosis are at highest risk of requiring revision surgery, either for continued craniofacial reconstruction due to progression of disease or for bony defects in or near the initial surgical site. These data give insight into risk of need for potential repeat surgeries depending on implant type and underlying diagnosis necessitating the implant and should be discussed with families when planning cranioplasty surgeries. Careful thought must be put into the various pros and cons of each implant type, with an individualized approach to implant choice when possible.

## Limitations

The major limitation in this retrospective review was the sample size of each group. While the total patient cohort included 149 patients, one of the largest cohorts from a single institution in current literature, over half of the patients underwent autologous cranioplasty. Several other implant types had low numbers, impacting the significance of evaluation of each alternative implant independently. Additionally, the distinction between autologous bone that was banked, stored in the abdominal cavity, or taken from a graft site was not made.

## Conclusion

Overall, this study adds to the current literature for pediatric cranioplasty. Alloplastic materials can be used in a pediatric cohort and may be considered even if autologous implant is available, particularly if infection is the underlying reason for cranioplasty.

## Statement of Ethics

This study protocol was reviewed and approved by the University of Florida IRB #201800653. IRB review granted an exemption for written consent for inclusion.

## Conflict of Interest Statement

The authors have no conflicts of interest to declare. Megan E.H. Still, Abraham Alvarado, Dan Neal, Lance S. Governale, and Jessica A. Ching were all employed by the University of Florida during the 3 years prior to obtaining data for chart review.

## Funding Sources

The authors have no sources of funding to report.

## Author Contributions

Megan E.H. Still and Sonja Samant collected data, wrote the manuscript, and Megan E.H. Still edited the final manuscript. Abraham Alvarado collected and analyzed original data and approved final manuscript. Dan Neal performed all statistical analysis. Lance S. Governale and Jessica A. Ching were responsible for study creation and editing/approving final manuscript.

## Data Availability Statement

Data were obtained via request from the University of Florida Integrated Data Repository from the electronic medical records and stored on a secured encrypted drive available to authors in HIPAA-compliant manner. All data generated or analyzed during this study are included in this article. Further inquiries can be directed to the corresponding author.

## Figures and Tables

**Table 1:** Independent factors as predictors of need for revision surgery after cranioplasty

	Overall (*N* = 149)	Revision surgery (*N* = 41, 27.5%)	*p* value
Gestational age at birth			
<37 weeks	9 (6.0)	3 (33.3)	0.707
≥37 weeks	140 (94.0)	38 (27.1)	
Age			
>6	104 (69.8)	23 (22.1)	**0.029**
≤6	45 (30.2)	18 (40.0)	
Gender			
Female	127 (85.2)	37 (29.1)	0.438
Male	22 (14.8)	4 (18.2)	
Diagnosis			
Craniosynostosis	39 (26.2)	17 (43.6)	**0.012**
Prior craniectomy or secondary skull defect	23 (15.4)	5 (21.7)	0.616
Infection	27 (18.1)	8 (29.6)	0.814
Trauma	36 (24.2)	9 (25.0)	0.831
Congenital bone defect	5 (3.4)	0 (0.0)	0.323
Tumor	13 (8.7)	2 (15.4)	0.516
Vascular lesion	6 (4.0)	0 (0.0)	0.188
Type of implant			
Autologous	96 (64.4)	34 (35.4)	**0.004**
PEEK	6 (4.0)	0 (0.0)	0.188
Titanium	13 (8.7)	1 (7.7)	0.114
Titanium + hydroxyapatite	4 (2.7)	1 (25.0)	1
PMMA	10 (6.7)	1 (10.0)	0.286
Hydroxyapatite	19 (12.8)	3 (15.8)	0.280
Acrylic	1 (0.7)	1 (100.0)	0.275

Categorical variables presented as *N* (% total patient cohort) in the overall column and as *N* (% of patient subpopulation) in the revision surgery column.

**Table 2:** Independent factors as predictors of need for removal of implant at time of revision surgery

	Overall (*N* = 149)	Removal of cranioplasty (*N* = 17, 11.4%)	*p* value
Gestational age at birth			
<37 weeks	9 (6.0)	2 (22.2)	0.273
≥37 weeks	140 (94.0)	15 (10.7)	
Age			
>6	104 (69.8)	10 (9.6)	0.399
≤6	45 (30.2)	7 (15.6)	
Gender			
Female	127 (85.2)	15 (11.8)	1
Male	22 (14.8)	2 (9.1)	
Diagnosis			
Craniosynostosis	39 (26.2)	8 (20.5)	0.074
Prior craniectomy or secondary skull defect	23 (15.4)	2 (8.7)	1
Infection	27 (18.1)	3 (11.1)	1
Trauma	36 (24.2)	3 (8.3)	0.764
Congenital bone defect	5 (3.4)	0 (0.0)	1
Tumor	13 (8.7)	1 (7.7)	1
Vascular lesion	6 (4.0)	0 (0.0)	1
Type of implant			
Autologous	96 (64.4)	14 (14.6)	0.115
PEEK	6 (4.0)	0 (0.0)	1
Titanium	13 (8.7)	1 (7.7)	1
Titanium + hydroxyapatite	4 (2.7)	1 (25.0)	0.387
PMMA	10 (6.7)	0 (0.0)	0.605
Hydroxyapatite	19 (12.8)	0 (0.0)	0.130
Acrylic	1 (0.7)	1 (100.0)	0.114

Categorical variables presented as *N* (% total patient cohort) in the overall column and as *N* (% patient subpopulation) in the removal of cranioplasty column.

## References

[1] Punchak M, Chung LK, Lagman C, Bui TT, Lazareff J, Rezzadeh K (2017). Outcomes following polyetheretherketone (PEEK) cranioplasty: systematic review and meta-analysis. J Clin Neurosci.

[2] Williams L, Fan K, Bentley R (2016). Titanium cranioplasty in children and adolescents. J Craniomaxillofac Surg.

[3] Artico M, Ferrante L, Pastore FS, Ramundo EO, Cantarelli D, Scopelliti D (2003). Bone autografting of the calvaria and craniofacial skeleton: historical background, surgical results in a series of 15 patients, and review of the literature. Surg Neurol.

[4] Ma IT, Symon MR, Bristol RE, Beals SP, Joganic EF, Adelson PD (2018). Outcomes of titanium mesh cranioplasty in pediatric patients. J Craniofac Surg.

[5] O'Reilly EB, Barnett S, Madden C, Welch B, Mickey B, Rozen S (2015). Computed-tomography modeled polyether ether ketone (PEEK) implants in revision cranioplasty. J Plast Reconstr Aesthet Surg.

[6] Abu-Ghname A, Banuelos J, Oliver JD, Vyas K, Daniels D, Sharaf B (2019). Outcomes and complications of pediatric cranioplasty: a systematic review. Plast Reconstr Surg.

[7] Zaccaria L, Tharakan SJ, Altermatt S (2017). Hydroxyapatite ceramic implants for cranioplasty in children: a single-center experience. Childs Nerv Syst.

[8] Frodel JL, Marentette LJ, Quatela VC, Weinstein GS (1993). Calvarial bone graft harvest. Techniques, considerations, and morbidity. Arch Otolaryngol Head Neck Surg.

[9] Grant GA, Jolley M, Ellenbogen RG, Roberts TS, Gruss JR, Loeser JD (2004). Failure of autologous bone-assisted cranioplasty following decompressive craniectomy in children and adolescents. J Neurosurg.

[10] Josan VA, Sgouros S, Walsh AR, Dover MS, Nishikawa H, Hockley AD (2005). Cranioplasty in children. Childs Nerv Syst.

[11] Blair GA, Gordon DS, Simpson DA (1980). Cranioplasty in children. Childs Brain.

[12] Klieverik VM, Miller KJ, Han KS, Singhal A, Vassilyadi M, Touchette CJ (2019). Cranioplasties following craniectomies in children-a multicenter, retrospective cohort study. Childs Nerv Syst.

[13] Bowers CA, Riva-Cambrin J, Hertzler DA, Walker ML (2013). Risk factors and rates of bone flap resorption in pediatric patients after decompressive craniectomy for traumatic brain injury. J Neurosurg Pediatr.

[14] Frassanito P, Massimi L, Caldarelli M, Tamburrini G, Di Rocco C (2012). Complications of delayed cranial repair after decompressive craniectomy in children less than 1 year old. Acta Neurochir.

[15] Frassanito P, Massimi L, Caldarelli M, Tamburrini G, Di Rocco C (2014). Bone flap resorption in infants. J Neurosurg Pediatr.

[16] Aloraidi A, Alkhaibary A, Alharbi A, Alnefaie N, Alaglan A, AlQarni A (2021). Effect of cranioplasty timing on the functional neurological outcome and postoperative complications. Surg Neurol Int.

[17] Morton RP, Abecassis IJ, Hanson JF, Barber JK, Chen M, Kelly CM (2018). Timing of cranioplasty: a 10.75-year single-center analysis of 754 patients. J Neurosurg.

[18] Xu H, Niu C, Fu X, Ding W, Ling S, Jiang X (2015). Early cranioplasty vs. late cranioplasty for the treatment of cranial defect: a systematic review. Clin Neurol Neurosurg.

